# Integrative development of a short screening questionnaire of highly processed food consumption (sQ-HPF)

**DOI:** 10.1186/s12966-021-01240-6

**Published:** 2022-01-24

**Authors:** Celia Martinez-Perez, Lidia Daimiel, Cristina Climent-Mainar, Miguel Ángel Martínez-González, Jordi Salas-Salvadó, Dolores Corella, Helmut Schröder, Jose Alfredo Martinez, Ángel M. Alonso-Gómez, Julia Wärnberg, Jesús Vioque, Dora Romaguera, José López-Miranda, Ramón Estruch, Francisco J. Tinahones, José Lapetra, Lluis Serra-Majem, Aurora Bueno-Cavanillas, Josep A. Tur, Vicente Martín Sánchez, Xavier Pintó, Miguel Delgado-Rodríguez, Pilar Matía-Martín, Josep Vidal, Clotilde Vázquez, Emilio Ros, Javier Basterra, Nancy Babio, Patricia Guillem-Saiz, María Dolores Zomeño, Itziar Abete, Jessica Vaquero-Luna, Francisco Javier Barón-López, Sandra Gonzalez-Palacios, Jadwiga Konieczna, Antonio Garcia-Rios, María Rosa Bernal-López, José Manuel Santos-Lozano, Maira Bes-Rastrollo, Nadine Khoury, Carmen Saiz, Karla Alejandra Pérez-Vega, María Angeles Zulet, Lucas Tojal-Sierra, Zenaida Vázquez Ruiz, Maria Angeles Martinez, Mireia Malcampo, José M. Ordovás, Rodrigo San-Cristobal

**Affiliations:** 1 Nutritional Genomics and Epigenomics Group, Precision Nutrition and Obesity Program, IMDEA Food, CEI UAM + CSIC, Ctra. Cantoblanco, 8, 28049 Madrid, Spain; 2 Biomedical Research Centre for Obesity Physiopathology and Nutrition Network (CIBEROBN), Instituto de Salud Carlos III (ISCIII), 28029 Madrid, Spain; 3 Department of Preventive Medicine and Public Health, University of Navarra, IdiSNA, 31009 Pamplona, Spain; 4 Department of Nutrition, Harvard T. H. Chan School of Public Health, Boston, MA 02115 USA; 5 Unitat de Nutrició Humana, Departament de Bioquímica i biotecnologia, Universitat Rovira i Virgili, 43201 Reus, Spain; 6 Human Nutrition Unit, Institut d’Investigació Sanitària Pere Virgili (IISPV), 43204 Reus, Spain; 7 Department of Preventive Medicine, University of Valencia, 46010 Valencia, Spain; 8 Cardiovascular Risk and Nutrition Research Group (CARIN), Hospital del Mar Medical Research Institute (IMIM), 08003 Barcelona, Spain; 9 Cardiometabolic Nutrition Group, IMDEA Food, CEI UAM + CSIC, 28049 Madrid, Spain; 10 Department of Nutrition, Food Sciences and Physiology, University of Navarra, 31009 Pamplona, Spain; 11 Bioaraba Health Research Institute, Osakidetza Basque Health Service, Araba University Hospital, University of the Basque Country UPV/EHU, 01009 Vitoria-Gasteiz, Spain; 12 Department of Nursing, School of Health Sciences, University of Málaga, Instituto de Investigación Biomédica de Málaga (IBIMA), 29016 Málaga, Spain; 13 Centro de Investigación Biomédica en Red de Epidemiología y Salud Pública (CIBERESP), Instituto de Salud Carlos III, 28029 Madrid, Spain; 14 Instituto de Investigación Sanitaria y Biomédica de Alicante (ISABIAL-UMH), 03010 Alicante, Spain; 15 Research Group on Nutritional Epidemiology & Cardiovascular Physiopathology (NUTRECOR), Health Research Institute of the Balearic Islands (IdISBa), University Hospital Son Espases (HUSE), 07120 Palma de Mallorca, Spain; 16 Lipids and Atherosclerosis Unit, Department of Internal Medicine, Maimonides Biomedical Research Institute of Cordoba (IMIBIC), Reina Sofia University Hospital, University of Cordoba, 14017 Córdoba, Spain; 17 Department of Internal Medicine, IDIBAPS, Hospital Clinic, University of Barcelona, 08007 Barcelona, Spain; 18 Department of Endocrinology, Instituto de Investigación Biomédica de Málaga (IBIMA), Virgen de la Victoria Hospital, University of Málaga, 29016 Málaga, Spain; 19 Department of Family Medicine, Research Unit, Distrito Sanitario Atención Primaria Sevilla, 41013 Sevilla, Spain; 20 Research Institute of Biomedical and Health Sciences (IUIBS), University of Las Palmas de Gran Canaria, Preventive Medicine Service, Centro Hospitalario Universitario Insular Materno Infantil (CHUIMI), Canarian Health Service, 35016 Las Palmas, Spain; 21 Department of Preventive Medicine and Public Health, University of Granada, 18011 Granada, Spain; 22 Research Group on Community Nutrition & Oxidative Stress, University of Balearic Islands-IUNICS & IDISBA, 07122 Palma de Mallorca, Spain; 23 Institute of Biomedicine (IBIOMED), University of León, 24071 León, Spain; 24 Lipids and Vascular Risk Unit, Internal Medicine, Hospital Universitario de Bellvitge, Hospitalet de Llobregat, 08907 Barcelona, Spain; 25 Departamento de Ciencias de la Salud, Centro de Estudios Avanzados en Olivar y Aceites de Oliva, Universidad de Jaén, 23071 Jaén, Spain; 26 Department of Endocrinology and Nutrition, Instituto de Investigación Sanitaria Hospital Clínico San Carlos (IdISSC), 28040 Madrid, Spain; 27 Biomedical Research Centre for Diabetes and Metabolic Diseases Network (CIBERDEM), Instituto de Salud Carlos III (ISCIII), 28029 Madrid, Spain; 28 Endocrinology and Nutrition Service, IDIBAPS, Hospital Clinic, University of Barcelona, 08007 Barcelona, Spain; 29 Department of Endocrinology and Nutrition, Hospital Fundación Jimenez Díaz, Instituto de Investigaciones Biomédicas IISFJD, University Autónoma, 28015 Madrid, Spain; 30 Department of Endocrinology and Nutrition, Complejo Hospitalario de Navarra, 31008 Pamplona, Spain; 31 School of Health Sciences, Blanquerna-Ramon Llull University, 08001 Barcelona, Spain; 32 Department of Public Health, University of Málaga, Instituto de Investigación Biomédica de Málaga (IBIMA), 29016 Málaga, Spain; 33 Internal Medicine Department, Regional University Hospital of Málaga, Instituto de Investigación Biomédica de Málaga (IBIMA), 29010 Málaga, Spain; 34 Nutrition and Genomics Laboratory, JM_USDA Human Nutrition Research Center on Aging, Tufts University, Boston, MA 02155 USA

**Keywords:** Ultra-processed food, Highly processed food, Questionnaire, PREDIMED-Plus, NOVA, Food processing-based classification

## Abstract

**Background:**

Recent lifestyle changes include increased consumption of highly processed foods (HPF), which has been associated with an increased risk of non-communicable diseases (NCDs). However, nutritional information relies on the estimation of HPF consumption from food-frequency questionnaires (FFQ) that are not explicitly developed for this purpose. We aimed to develop a short screening questionnaire of HPF consumption (sQ-HPF) that integrates criteria from the existing food classification systems.

**Methods:**

Data from 4400 participants (48.1% female and 51.9% male, 64.9 ± 4.9 years) of the Spanish PREDIMED-Plus (“PREvention with MEDiterranean DIet”) trial were used for this analysis. Items from the FFQ were classified according to four main food processing-based classification systems (NOVA, IARC, IFIC and UNC). Participants were classified into tertiles of HPF consumption according to each system. Using binomial logistic regression, food groups associated with agreement in the highest tertile for at least two classification systems were chosen as items for the questionnaire. ROC analysis was used to determine cut-off points for the frequency of consumption of each item, from which a score was calculated. Internal consistency of the questionnaire was assessed through exploratory factor analysis (EFA) and Cronbach’s analysis, and agreement with the four classifications was assessed with weighted kappa coefficients.

**Results:**

Regression analysis identified 14 food groups (items) associated with high HPF consumption for at least two classification systems. EFA showed that items were representative contributors of a single underlying factor, the “HPF dietary pattern” (factor loadings around 0.2). We constructed a questionnaire asking about the frequency of consumption of those items. The threshold frequency of consumption was selected using ROC analysis. Comparison of the four classification systems and the sQ-HPF showed a fair to high agreement. Significant changes in lifestyle characteristics were detected across tertiles of the sQ-HPF score. Longitudinal changes in HPF consumption were also detected by the sQ-HPF, concordantly with existing classification systems.

**Conclusions:**

We developed a practical tool to measure HPF consumption, the sQ-HPF. This may be a valuable instrument to study its relationship with NCDs.

**Trial registration:**

Retrospectively registered at the International Standard Randomized Controlled Trial Registry (ISRCTN89898870) on July 24, 2014.

**Supplementary Information:**

The online version contains supplementary material available at 10.1186/s12966-021-01240-6.

## Background

Changes in eating patterns are occurring worldwide [1]. A common feature of such changes is the transition from minimally processed food to moderately processed to highly processed (HPF) or ultra-processed foods (UPF) [2–10]. A widely used, although controversial [11], definition of UPF is that these are “industrial formulations made mostly or entirely from substances derived from foods and additives, with little if any intact food” [12]. According to the NOVA classification system, these foods are highly palatable and habit-forming, microbiologically safe, affordable, strongly marketed and advertised, and sold in convenient and attractive packaging, promoting their overconsumption [12, 13]. This, together with the evidence showing their negative impact on health [9, 10, 14–18], has turned UPF consumption into a potential public health concern [19] in need of more solid research. While the term UPF is mainly attributed to the NOVA system, other food processing-based classification systems have described foods and drinks of similar characteristics under their categories of HPF [20, 21], so we will use the term HPF to refer to this type of foods.

Although numerous studies have demonstrated a link between the risk of developing non-communicable diseases and HPF consumption [12, 17, 22–27], epidemiological research faces some hurdles in this field. First, the existence of multiple food processing-based classification systems based on different criteria [21, 28] results in heterogeneous conclusions regarding health outcomes depending on the system used, as shown recently for cardiometabolic health markers [20]. Second, the lack of an effective tool to assess HPF consumption in clinical studies severely hinders the downstream analysis of its relationship to disease risk. For instance, in the PREDIMED-Plus (from the Spanish “PREvention with MEDiterranean DIet”) trial, dietary intake was assessed through a lengthy food frequency questionnaire (FFQ). Therefore, estimation of HPF consumption requires the classification of FFQ items according to the selected classification system, as previously done [20, 24, 25, 29]. This process is time-consuming and subject to bias on the part of the researcher classifying the items since FFQs are not specifically designed to include HPF. This is because an FFQ is designed to estimate consumption of general commonly consumed foods, of which some may fall into the HPF category, but this depends on the classification system used [20]. In addition, calculations of daily consumption of each HPF item and percentage over total intake (in grams or kcal per day) are commonly used to infer their association with health outcomes [9, 17, 30]. These calculations are not direct and time-consuming when derived from current tools such as FFQs or 24 h recalls. There is, therefore, the need for an easy-to-use and comprehensive measure that assesses HPF consumption in the general population. This paper describes the development of a new screening questionnaire that allows an easy and quick determination of a subject’s HPF consumption, the sQ-HPF. We view this as an integrative tool since it incorporates criteria from four food-processing-based classification systems. We developed this questionnaire based on available data from an FFQ to create a tool that could replace the estimation of HPF consumption from FFQ in future studies. We hypothesize that the sQ-HPF is comparable in terms of evaluating HPF consumption to other dietary assessment tools that must be used in combination with food processing-based classification systems and can effectively capture longitudinal changes in HPF consumption.

This questionnaire will be of potential interest to the scientific community, especially in the context of clinical nutrition and public health. Its use should minimize the difficulty in comparing results from studies that use different classification systems, allowing a straightforward evaluation of the HPF dietary pattern in large epidemiological studies efficiently and comparably. Altogether, it will enable the development of tailored nutritional interventions and food policies to limit HPF consumption and prevent diet-related diseases.

## Methods

### Study population

**Fig. 1:**
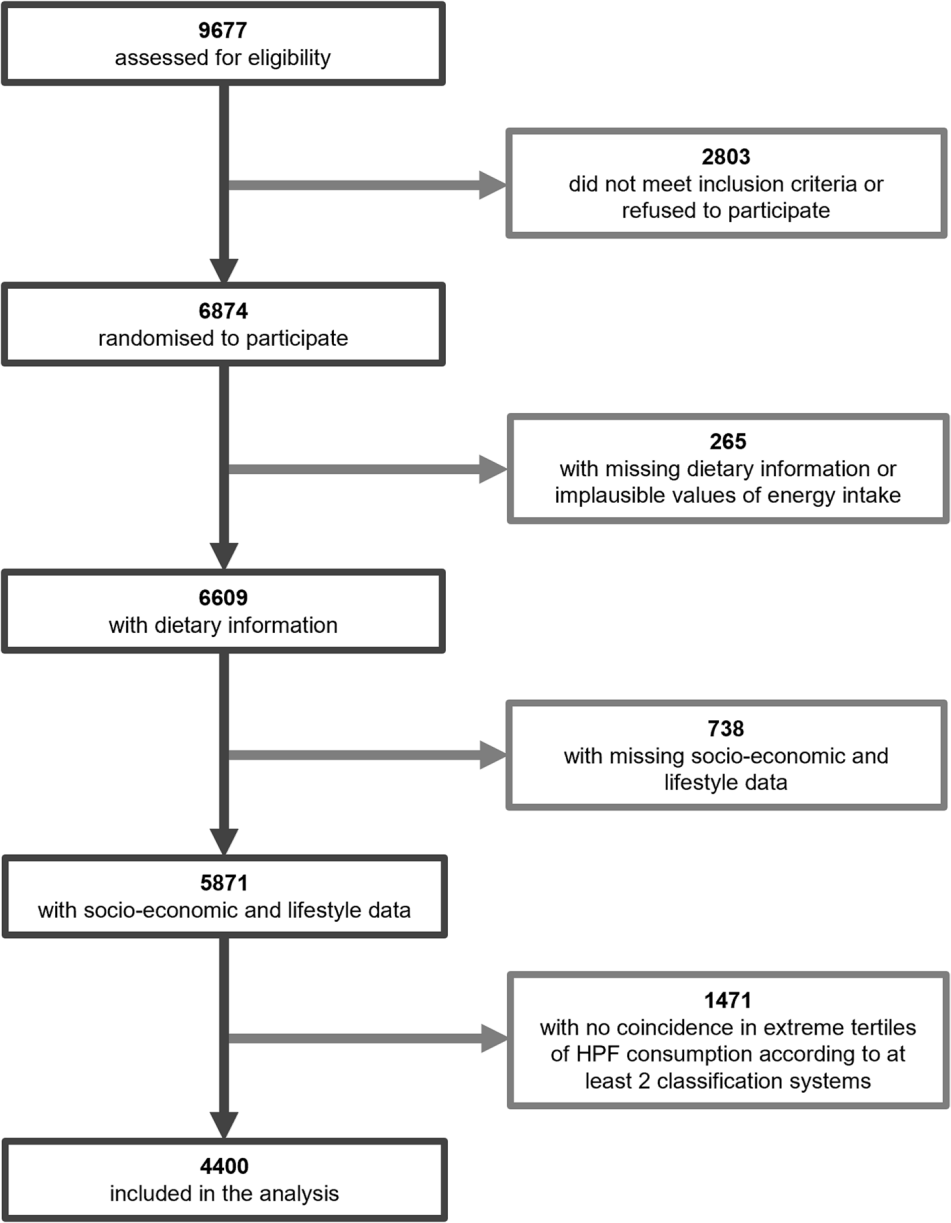
Flowchart of the PREDIMED-Plus participants. Number of subjects shown in bold

Data from the PREDIMED-Plus trial (from the Spanish “PREvention with MEDiterranean DIet”) was used. This is an ongoing 6-year, multicenter, randomized, parallel-group clinical trial launched in Spain in 2013. The main aim of the study is to evaluate the effect on primary cardiovascular disease prevention of an intensive weight loss and its long-term maintenance through a lifestyle intervention based on three pillars: energy-restricted Mediterranean diet (er-MedDiet), increased physical activity (PA) and behavioral support. The study protocol, including study design and data collection, can be found at the PREDIMED-Plus website (https://www.predimedplus.com/en/) and was approved according to the ethical standards of the Declaration of Helsinki by the Institutional Review Boards (IRBs) of all participating centers. All participants provided written consent for their participation in the study. The trial is conducted in 23 Spanish centers and involves 6874 participants (48.5% female, 54.5% male) between 55 and 75 years old (mean age and SD 65.0 ± 4.9) who presented with overweight or obesity (BMI ≥ 27 and < 40 kg/m^2^) and met at least three criteria for metabolic syndrome (MetS) as previously described [31]. Details about the cohort have been described elsewhere [32]. The trial was retrospectively registered at the International Standard Randomized Controlled Trial Registry with number 89898870 on 24th July 2014. The present analysis used baseline and longitudinal (years 1 and 2) data from the PREDIMED-Plus study data set dated 26th June 2020 was used. Participants with implausible energy intakes (< 500 or > 3500 kcal for females and < 800 or > 4000 kcal for males) were excluded. In addition, participants with missing values for dietary, lifestyle and socioeconomic variables were not included in the analysis. After the definition of the tertile agreement variable (see Statistical analyses section), participants with (1) no coincidence in extreme tertiles of HPF consumption (either in tertile 1 or tertile 3) according to at least two classification systems and (2) participants that were classified in tertile 1 for two classification systems and tertile 3 for the other two classification systems were excluded. This was done to select extreme HPF consumers to further construct the binomial regression model. The final number of participants included in the analysis for the development of the questionnaire was 4400 (48.1% female and 51.9% male, 64.9 ± 4.9 years) (Fig. 1). This study adhered to the STROBE-nut reporting guidelines [33].

### Blood measurements

Trained nurses collected blood samples after overnight fasting at the recruiting centers or primary health care centers. Plasma glucose, triglycerides and total cholesterol levels were measured following standard enzymatic methods.

### Anthropometric measurements

Weight and waist circumference measurements were taken from participants in light clothing with no shoes or accessories, using an electronic calibrated scale and an anthropometric tape, respectively. Waist circumference was measured midway between the lowest rib and the iliac crest. Height measurements were taken using a wall-mounted stadiometer. Body Mass Index (BMI) was calculated as the weight in kilograms divided by the square of height in meters.

### Lifestyle measurements

Socioeconomic and PA data were collected through the general PREDIMED-Plus questionnaire. Based on the validated questionnaires, the REGICOR [34] and the Rapid Assessment of Physical Activity (RAPA) [35], participants were asked about the frequency and intensity of physical activities, and three levels of PA were defined as follows: low (frequent sitting and little walking and/or frequent sitting and moderate sustained efforts), medium (frequent walking with no vigorous efforts), high (frequent walking and vigorous efforts and/or frequent vigorous efforts). Sedentary behaviors were assessed through a validated questionnaire, the Spanish version of the Nurses’ Health Study (NHS) questionnaire [36]. Data about eating habits (binge eating, snacking) were collected within the multidimensional scale of weight locus control questionnaire [37]. Trained interviewers administered questionnaires in individual face-to-face sessions.

### Dietary measurements

To assess the dietary intake of participants over the last year, a validated semi-quantitative 143-item FFQ [38–40] that considers variations in dietary patterns among seasons, weekdays and weekends was used. Participants were asked the average frequency of consumption of a commonly used portion size (e.g., glass, cup, slice) for each food or beverage item. Nine options for frequency of consumption are given, ranging from “never or hardly ever” to “more than six times a day.” To estimate the daily consumption for each item, the portion size was multiplied by the frequency of consumption and then expressed as grams per day. This calculation was not possible for the fried foods item as the portion size is not specified in the FFQ. To assess adherence to an er-MedDiet, a 17-item questionnaire specially developed and validated for the PREDIMED-Plus trial [41, 42] was used. Through this questionnaire, the frequency of consumption of traditional Mediterranean food items is evaluated. One point is scored when the answer meets specific criteria defining er-MedDiet, so the higher the score, the better adherence to this diet.

### HPF consumption and food processing-based classification systems

Four food-processing based classification systems – the NOVA [12, 43, 44], the International Agency for Research on Cancer (IARC) [7, 45], the International Food Information Council Foundation (IFIC) [46, 47], and the University of North Carolina (UNC) [48] systems – were used to classify FFQ items into processing categories as previously described [20]. In the present study, HPF refers to the following groups: Group 4 for NOVA, Group 3 for IARC, Groups 4 and 5 for IFIC, and Groups 4.1 and 4.2 for UNC. For each participant, HPF consumption was estimated according to each classification system as the sum of grams per day consumed from foods in the HPF group, divided by the total grams of food consumed per day and multiplied by 100 [20]. The frequency of consumption was directly obtained from FFQ answers in times per day.

### Statistical analyses

Data analysis was conducted using R programming language [49] in RStudio [50] and with the following statistical packages: “DescTools” [51], “psych” [52], “tableone” [53], “cutpointr” [54], “corrplot” [55], “vcd” [56] and rstatisx [57]. Participants were classified according to tertiles of HPF consumption for each classification system (T1 – low HPF consumption, T3 high HPF consumption). Tertiles were chosen to capture and show the variability in HPF consumption while allowing a straightforward comparison between classification systems. Then, subjects were classified according to tertile agreement for, at least, two classification systems. Those classified in T3 in at least two classification systems scored “1” for tertile agreement, while those classified in T1 for at least two classification systems scored “0”. FFQ items were classified into food groups according to their similar nature, nutritional characteristics, and/or form of consumption (Supplementary Table 1). The association between tertile agreement and frequency of consumption for each food group was analyzed through binomial logistic regression, adjusted for age, sex, recruitment center, energy intake, physical activity level, medication for blood pressure and diabetes, working status, educational level, and civil status, using the “glm” function from R base package “stats.” These covariates were selected due to their potential effect on HPF consumption. To calculate optimal cut-off points of frequency of consumption for the selected items, receiver operating characteristic (ROC) analysis was performed using the R package “cutpointr” and the “cutpointr” function [54]. The method to estimate the cut-off points was based on the maximization of the Youden-Index [58]. Estimated cut-off points were used to establish the criteria for scoring 1 point in the sQ-HPF, which indicated high HPF consumption, or 0, indicating low HPF consumption. Cut-off points were adapted to the nine possible answers of the FFQ, so the criteria for scoring were based on a threshold frequency of consumption for each item (i.e., food group). In parallel, exploratory factor analysis (EFA) was performed to identify underlying relationship patterns between items included in the sQ-HPF using the “fa” function from the “psych” package [52]. To test for data suitability for the EFA, the Kaiser-Meyer-Olkin Criterion [59] and Bartlett’s test of sphericity [60] were applied. The EFA was performed without rotation and with a principal factor solution as a factoring method. Factor retention was based on the scree plot and Kaiser’s criterion [61]. Cronbach’s alpha [62] was calculated as a measure of internal consistency of the questionnaire. Using the criteria for scoring, the sQ-HPF score was calculated for each subject in the PREDIMED-Plus database, and the questionnaire estimated HPF consumption was calculated through linear regression analysis using the sQ-HPF score as the dependent variable. For descriptive analyses, participants were classified into tertiles of the sQ-HPF score. Data is shown in tables as “mean (standard deviation, SD)” for continuous variables and as “number of subjects (%)” for categorical variables. Statistically significant differences (*p* < 0.05) in dietetic and lifestyle variables among tertiles were compared using a one-way ANOVA test for continuous variables and a Chi-squared test for categorical variables. *P*-values were adjusted for age, sex, recruitment center, energy intake, physical activity level, medication for blood pressure and diabetes, working status, educational level, and civil status. To assess the concordance between tertiles of HPF consumption calculated by the four classification systems and by the sQ-HPF, weighted Cohen’s kappa (κ) coefficients were calculated with the function “Kappa” from the R package “vcd”. For longitudinal analysis of HPF consumption, a linear mixed model was performed using the R package “lme4” and “emmeans” with the same covariates as previous analyses. The number of subjects used for this analysis was 3284 due to longitudinal data loss.

## Results

### General characteristics of the PREDIMED-Plus cohort according to tertile agreement

Subjects scored 1 in the tertile agreement variable if they were classified in the highest tertile (T3) of HPF consumption for at least two classifications systems, while they scored 0 if they were classified in the lowest tertile (T1) of HPF consumption for at least two classifications systems. General characteristics of PREDIMED-Plus participants at baseline according to the scores of the tertile agreement variable are shown in Table 1. Subjects who scored 1 (high HPF consumption by at least two classification systems) were mainly men (73.1%), had higher energy intake (2559.84 kcal/day) and lower MedDiet adherence (7.65 points) than subjects who scored 0 (low HPF consumption by at least two classifications systems). Among the high HPF subjects, 78.8% were married and 41.8% had a primary education level. Around half of the subjects who scored 1 showed a low level of PA (55.3%) and were taking medication for cholesterol (49.8%). In addition, 75.7% of them were taking medication for blood pressure and 25.9% were on diabetes medication. According to these results, the variables age, sex, recruitment center, energy intake, physical activity level, medication for blood pressure and diabetes, working status, educational level, and civil status were selected as covariates for further analysis due to their potential effect on HPF consumption. MedDiet adherence was not selected as a covariate due to the presence of collinearity with HPF consumption.

**Table 1:** General characteristics of PREDIMED-Plus participants at baseline according to tertile agreement

	Tertile agreement^a^		
	Low HPF	High HPF	p
**n**	2186	2214	
**Age (years)**	66.14 (4.50)	63.68 (5.07)	**< 0.001**
**Female sex (%)**	1522 (69.6)	595 (26.9)	**< 0.001**
**Civil status (%)**			**0.001**
Single	115 (5.3)	109 (4.9)	
Married	1622 (74.2)	1745 (78.8)	
Widowed/divorced	449 (20.5)	360 (16.3)	
**Education level (%)**			
Primary	1252 (57.3)	926 (41.8)	**< 0.001**
Secondary	538 (24.6)	704 (31.8)	
College	396 (18.1)	584 (26.4)	
**Active working status (%)**	307 (14.0)	642 (29.0)	**< 0.001**
**Energy intake (kcal/day)**	2158.46 (480.16)	2559.84 (571.11)	**< 0.001**
**MedDiet adherence score**	9.46 (2.54)	7.65 (2.61)	**< 0.001**
**PA level (%)**			**0.001**
Low	1188 (54.3)	1225 (55.3)	
Medium	904 (41.4)	841 (38.0)	
High	94 (4.3)	148 (6.7)	
**Blood pressure medication (%)**	1717 (78.5)	1676 (75.7)	**0.027**
**Cholesterol medication (%)**	1123 (51.4)	1102 (49.8)	0.303
**Diabetes medication (%)**	631 (28.9)	573 (25.9)	**0.029**

Data shown as “mean (standard deviation, SD)” for continuous variables and as “number of subjects (%)” for categorical variables. One-way ANOVA test used for continuous variables and Chi-squared test used for categorical variables. Significant *p*-values (< 0.05) shown in bold

*MedDiet* Mediterranean diet, *PA* Physical activity

^a^Tertile agreement variable: score 0 – “low HPF consumer” if a subject is classified in T1 of HPF consumption by at least two classification systems; score 1 – “high HPF consumer” if a subject is classified in T3 by at least two classification systems. HPF: highly processed food. *N* = 4400

### Development of the sQ-HPF

Food groups were defined based on the similarity in nature, nutritional profile and/or form of consumption of the PREDIMED-Plus FFQ food and beverage items (Supplementary Table 1). Food groups chosen to be included in the sQ-HPF showed a significant positive association (p-value adjusted by Bonferroni < 0.05) between their frequency of consumption and a value of 1 of tertile agreement, i.e., the subject is in tertile 3 of HPF consumption for at least two food processing-based classification systems (Table 2). A final solution of 14 food groups was selected and included the following: fatty dairy products, sugary dairy products, cured meat, fats, fermented alcohol, distilled alcohol, sugary and artificially sweetened drinks, sweets, snacks, ready to eat products, refined cereals, sauces, additives, and fried foods.

**Table 2:** Associations between candidate sQ-HPF items and tertile agreement variable by binomial logistic regression

Predictor variable	β	SE	p (Bonf)
**Fatty dairy products**	0.33	0.10	**0.024**
**Sugary dairy products**	1.37	0.22	**< 0.001**
**Cured meat**	0.59	0.07	**< 0.001**
**Fats**	0.63	0.14	**0.001**
**Fermented alcohol**	1.59	0.07	**< 0.001**
**Distilled alcohol**	3.90	0.45	**< 0.001**
**Sugary drinks**	3.90	0.17	**< 0.001**
**Sweets**	0.46	0.05	**< 0.001**
**Snacks**	3.93	0.43	**< 0.001**
**Ready To Eat (RTE)**	2.91	0.33	**< 0.001**
**Refined cereals**	0.30	0.04	**< 0.001**
**Sauces**	0.97	0.18	**< 0.001**
**Additives**	0.10	0.02	**< 0.001**
**Fried food**	0.84	0.15	**< 0.001**
Whole dairy products	−0.01	0.06	1
Semi-skimmed dairy products	− 0.15	0.03	**< 0.001**
Eggs	−0.31	0.19	1
Red meat	0.05	0.15	1
White meat	−1.41	0.17	**< 0.001**
White fish	−1.40	0.16	**< 0.001**
Blue fish	−1.29	0.15	**< 0.001**
Vegetables	−0.55	0.03	**< 0.001**
Fruit	−1.01	0.04	**< 0.001**
Potatoes	−0.88	0.18	**< 0.001**
Nuts	−0.87	0.07	**< 0.001**
Legumes	−2.46	0.23	**< 0.001**
Oils	−0.21	0.03	**< 0.001**
Non-sugary drinks	−0.09	0.03	0.302
Wholegrain cereals	−0.53	0.04	**< 0.001**
Vitamin/supplements	−0.10	0.12	1
Binge eating	0.17	0.14	1
N° binges/week	0.05	0.05	1
Snacking	0.06	0.08	1

Binomial logistic regression adjusted for age, sex, recruitment center, energy intake, physical activity level, medication for blood pressure and diabetes, working status, educational level, and civil status. Tertile agreement is the outcome variable (score 0 – “low HPF consumer” if a subject is classified in T1 of HPF consumption by at least two classification systems; score 1 – “high HPF consumer” if a subject is classified in T3 by at least two classification systems). Food groups expressed in frequency of consumption (times/day). HPF: highly processed food. Items selected for the sQ-HPF are shown in bold. Bonf: Bonferroni adjustment. *N* = 4400

EFA revealed that most of the items selected for the sQ-HPF had factor loadings higher than 0.2, indicating that they were representative contributors to the factor (Table 3). The measure of sample adequacy (MSA) was 0.78, considered as “good” for the verification of the proportion of variance in variables that can be caused by factors, according to the Kaiser-Meyer-Olkin Criterion. Bartlett’s test of sphericity was highly significant (*p* < 0.001), indicating that variables were correlated in the population. This, together with the MSA value, indicated the adequacy of the data to proceed with EFA. Factor retention applying the Kaiser criterion revealed a single underlying factor being identified by the questionnaire items, namely the HPF dietary pattern. The internal consistency of the questionnaire items was evaluated with Cronbach’s alpha, which had a moderate value of 0.67.

**Table 3:** Exploratory factor analysis for sQ-HPF items

Factor 1: HPF diet	Factor loadings
Ready To Eat (RTE)	0.45
Fried food	0.44
Fermented alcohol	0.42
Snacks	0.42
Fatty dairy products	0.38
Sugary drinks	0.37
Distilled alcohol	0.36
Sauces	0.35
Refined cereals	0.35
Cured meat	0.34
Sweets	0.3
Sugary dairy products	0.29
Additives	0.26
Fats	0.23

Bartlett’s test of sphericity = *p* < 0.001

Measure of Sample Adequacy (MSA) = 0.78

Standardized Cronbach’s α = 0.67

*N* = 4400

The sQ-HPF was based on the 14 food groups selected previously. Each item asked about the frequency of consumption of a particular food group (Table 4). Examples of representative food and beverage items included in each food group were provided for each item. Estimated cut-off points were used to determine the threshold frequency of consumption to consider the respondent as an HPF consumer for the item, as shown in the column “Criteria for 1 point” in Table 4. For the self-reported version of the questionnaire, this column should be removed from the questionnaire form since it is intended for the use of the person assessing the score only. Using baseline data from the PREDIMED-Plus FFQ, the percentage of HPF over total grams per day according to the questionnaire items was calculated for each patient. This was used as the dependent variable in a linear regression with the sQ-HPF score obtained for each patient to establish the following equation for the regression line: “HPF consumption (% g/day) = (3.7 x sQ-HPF score) + 7.6”. This equation allows the estimation of the HPF consumption from the sQ-HPF score, as shown at the bottom of Table 4.

**Table 4:** Screening questionnaire of highly processed food consumption (sQ-HPF)

**What was your average frequency of consumption over the past year of…**										**Criteria for 1 point**			**Score** **(0 or 1)**	
**Q1. Fatty dairy products** (cream, cured or semi-cured cheese, processed soft cheese wedges)?										> = 2 t/week				
**Q2. Sugary dairy products** (condensed milk, industrially produced milkshakes, flavored Petit Suisse yogurt, custard, crème caramel flan, pudding)?										> 3 t/month				
**Q3. Cured meats** (serrano ham, sandwich (deli) ham, cured cold meats, pâté, bacon, marbling)?										> = 1 t/day				
**Q4. Fats** (margarine, butter, lard (animal fat))?										> 3 t/month				
**Q5. Fermented alcohols** (rosé wine, muscatel wine, young red wine, aged red wine, white wine, Spanish sparkling wine (cava), beer)?										> 1 t/day				
**Q6. Distilled alcohols** (liquors, anisette, whisky, gin, vodka, cognac)?										> 3 t/month				
**Q7. Sugary and artificially sweetened drinks** (soft drinks, artificially sweetened drinks, bottled juice, grape must)?										> = 2 t/week				
**Q8. Sweets** (ice-cream and sorbets, canned fruit in juice or syrup, biscuits, whole meal biscuits, chocolate biscuits, honey, homemade baking products, industrially produced confectionery, donuts, muffins, cupcakes, industrially produced cakes, churros, chocolates, soluble cocoa powder, nougat, marzipan, shortbread biscuits, jam)?										> 1 t/day				
**Q9. Snacks** (packaged potato crisps/chips, packaged snacks)?										> 3 t/month				
**Q10. Ready To Eat products** (pizza, croquettes, instant soup)?										> 3 t/month				
**Q11. Refined cereals** (white and sliced bread, breakfast cereals, Spaghetti, macaroni, Spanish noodles, white rice)?										> = 2 t/week				
**Q12. Sauces** (mustard, mayonnaise, tomato sauce, Ketchup)?										> 1 t/week				
**Q13. Additives** (sugar, table salt)?										> 3 t/day				
**Q14. Fried foods** (eat out and homemade)?										> = 2 t/week				
										**TOTAL SCORE:**				
Equivalency between sQ-HPF score and the estimated percentage of HPF consumption over the total intake in grams per day:														
**Score**	1	2	3	4	5	6	7	8	9	10	11	12	13	14
**% HPF**	11.3	15	18.7	22.4	26.1	29.8	33.5	37.2	40.9	44.6	48.3	52	55.7	59.4

**Fig. 2:**
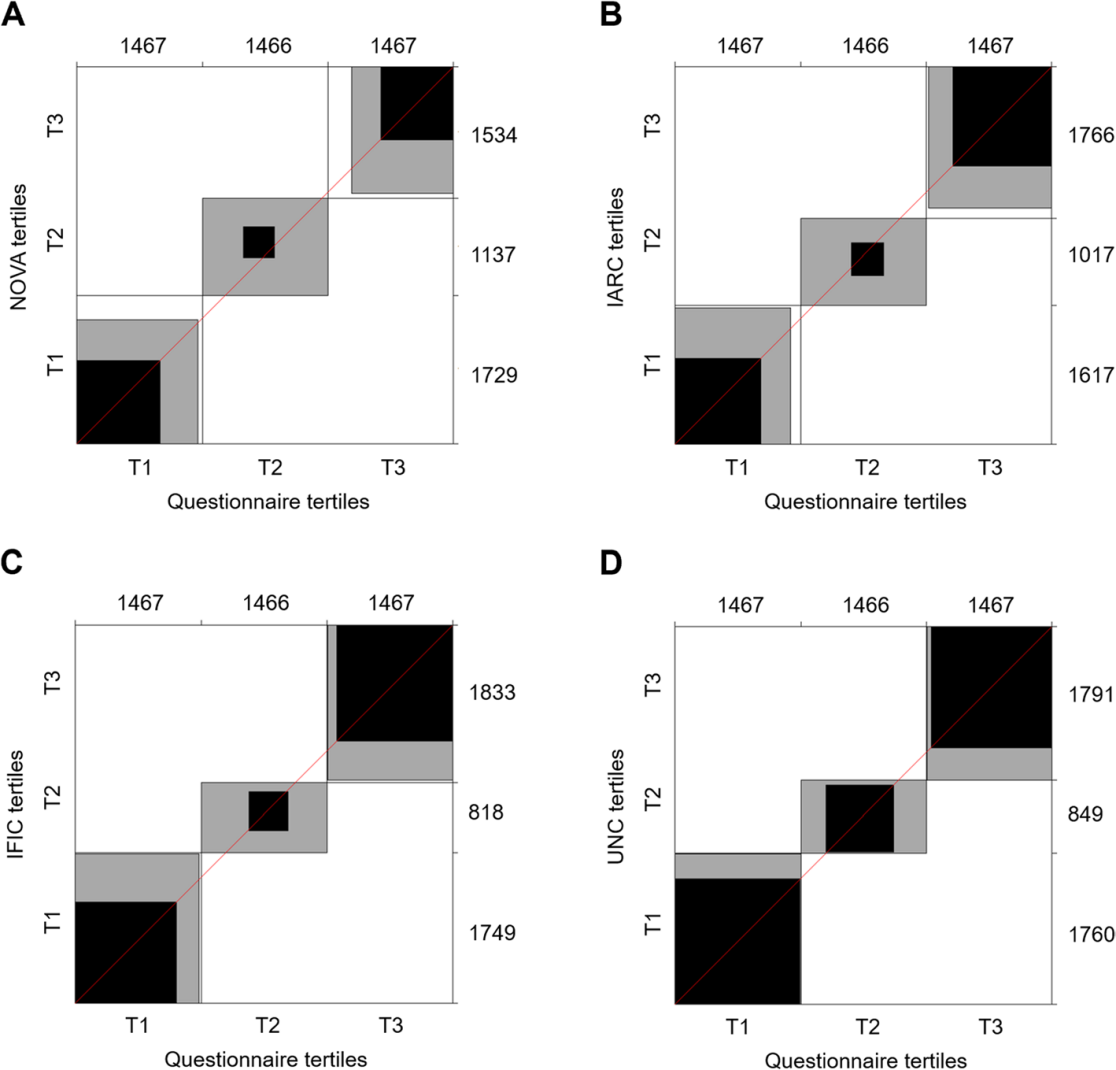
Agreement plots of tertiles of HPF consumption. Visual representation of contingency tables between the sQ-HPF tertiles and **A** NOVA HPF tertiles, **B** IARC HPF tertiles, **C** IFIC HPF tertiles and **D** UNC HPF tertiles. Marginal totals of the contingency table are located on the top and right axis. Shading represents the level of agreement, black indicates “perfect agreement”, and grey indicates “partial agreement”. The extent to which rectangles deviate from the diagonal line of no bias indicates the extent of disagreement, and the position (above/below) indicates direction of the disagreement. HPF: highly processed food

Weighted κ coefficients were calculated between HPF consumption tertiles according to the sQ-HPF and the four existing classification systems (Supplementary Table 2). The highest agreement was for the comparison with UNC tertiles (κ = 0.88), followed by IFIC tertiles (κ = 0.65) and IARC tertiles (κ = 0.50). The comparison of tertiles according to the sQ-HPF with NOVA tertiles of HPF showed a fair agreement (κ = 0.36). These comparisons were in accordance with the corresponding agreement plots (Fig. 2).

### Dietary, lifestyle and cardiometabolic characteristics of PREDIMED-Plus participants according to the sQ-HPF score

We aimed to investigate whether changes in HPF consumption as estimated from the FFQ and the four different systems were also detected when the sQ-HPF was used. In addition, we wanted to analyze the dietary profile across tertiles of the sQ-HPF score, since this is a tool to evaluate a particular dietary pattern. Dietary characteristics of PREDIMED-Plus participants at baseline are shown in Table 5**,** grouped by HPF consumption tertiles according to the score obtained in the sQ-HPF (T1 – lowest score, T3 – highest score). Consumption of food groups included in the questionnaire was the highest for subjects in T3 of the sQ-HPF score, which was not the case for vegetables, fruits, and legumes. Dairy products and fish consumption were not different among tertiles. With all the classification systems, subjects in T3 showed the highest HPF consumption (percentage over total grams per day: 12.45 ± 8.51 for NOVA, 54.85 ± 11.01 for IARC, 29.26 ± 12.02 for IFIC and 30.34 ± 12.40 for UNC, 35.05 ± 12.24 for questionnaire estimated HPF consumption).

**Table 5:** Dietetic characteristics of PREDIMED-Plus participants at baseline according to tertiles of the sQ-HPF score

	Tertiles of the sQ-HPF score			
	T1 (lowest)	T2	T3 (highest)	p^1^
**n**	1765	1223	1412	
**sQ-HPF score**	1.99 (0.96)	4.49 (0.50)	7.49 (1.47)	**< 0.001**
**Quest. HPF (% of g/day)**	13.91 (9.13)	25.45 (12.73)	35.01 (12.24)	**< 0.001**
**NOVA HPF (% of g/day)**	4.86 (5.04)	8.37 (7.23)	12.45 (8.51)	**< 0.001**
**IARC HPF (% of g/day)**	38.85 (11.36)	47.47 (12.34)	54.85 (11.01)	**< 0.001**
**IFIC HPF (% of g/day)**	13.17 (8.85)	21.77 (12.33)	29.26 (12.02)	**< 0.001**
**UNC HPF (% of g/day)**	11.40 (8.82)	21.79 (12.83)	30.34 (12.40)	**< 0.001**
**Fatty dairy (g/day)**	11.13 (13.93)	19.15 (21.36)	25.19 (20.27)	**< 0.001**
**Sugary dairy (g/day)**	3.47 (13.47)	7.09 (21.96)	16.46 (36.18)	**< 0.001**
**Cured meats (g/day)**	24.20 (16.27)	32.75 (21.80)	44.62 (27.46)	**< 0.001**
**Fats (g/day)**	0.66 (2.14)	1.47 (3.47)	2.33 (4.18)	**< 0.001**
**Fermented alcohol (g/day)**	73.34 (149.00)	208.30 (298.60)	324.26 (342.83)	**< 0.001**
**Distilled alcohol (g/day)**	0.80 (4.37)	3.23 (11.13)	7.74 (17.03)	**< 0.001**
**Sugary drinks (g/day)**	33.95 (97.29)	77.08 (153.91)	143.91 (195.14)	**< 0.001**
**Sweets (g/day)**	32.99 (30.54)	53.02 (47.10)	65.94 (43.71)	**< 0.001**
**Snacks (g/day)**	1.27 (3.05)	2.90 (5.85)	6.65 (10.15)	**< 0.001**
**Ready To Eat (g/day)**	3.81 (6.73)	7.18 (12.61)	12.99 (16.23)	**< 0.001**
**Refined cereals (g/day)**	71.23 (65.58)	110.87 (88.72)	146.36 (89.88)	**< 0.001**
**Sauces (g/day)**	1.56 (2.12)	2.52 (2.77)	4.15 (4.42)	**< 0.001**
**Additives (g/day)**	5.04 (8.08)	8.53 (11.67)	13.45 (14.90)	**< 0.001**
**Fried (times/day)**	0.14 (0.17)	0.28 (0.33)	0.43 (0.37)	**< 0.001**
**Vegetables (g/day)**	362.07 (148.70)	324.10 (142.63)	296.94 (131.27)	**< 0.001**
**Fruits (g/day)**	397.36 (226.62)	354.80 (215.93)	309.72 (194.70)	**< 0.001**
**Legumes (g/day)**	21.06 (12.03)	21.18 (12.41)	20.12 (10.04)	**0.018**
**Cereals (g/day)**^a^	125.72 (64.90)	149.96 (80.81)	174.99 (80.06)	**< 0.001**
**Dairy (g/day)**	342.27 (210.61)	337.07 (201.45)	346.82 (202.03)	0.428
**Meat (g/day)**	131.87 (53.71)	146.04 (56.49)	166.94 (61.44)	**< 0.001**
**Fish (g/day)**	103.97 (46.74)	101.91 (46.44)	101.30 (50.20)	0.211

Data shown as “mean (standard deviation, SD)”. One-way ANOVA test used for continuous variables. Significant *p*-values (< 0.05) shown in bold. HPF consumption was estimated as the percentage over total grams per day, except for fried foods (data not available)

^1^*P*-values adjusted for age, sex, recruitment center, energy intake, physical activity level, medication for blood pressure and diabetes, working status, educational level, and civil status

^a^Excluding potatoes. MedDiet: Mediterranean Diet. *N* = 4400

Associations between lifestyle and cardiometabolic parameters and HPF consumption have been previously reported, so we next aimed to analyze if we could detect changes in these variables across tertiles of the sQ-HPF score. Lifestyle and cardiometabolic characteristics of PREDIMED-Plus participants at baseline grouped by HPF consumption tertiles showed differences according to the sQ-HPF score (Table 6). Participants ranked in the highest tertile had higher levels of triglycerides (161.05 ± 90.55 mg/dL), higher weight (90.68 ± 13.10 kg) and waist circumference (109.83 ± 9.45 cm) compared to those in the lowest tertile. No significant changes among tertiles were detected in fasting glucose and total cholesterol levels. Subjects in T3 of the sQ-HPF score spent more time watching TV than T1 subjects (4.05 ± 2.02 h/day), while sleeping hours were similar across tertiles. Around half of the subjects classified in T3 were classified as sedentary (51.5%). Concerning eating habits, 30.5% of the subjects with the highest sQ-HPF score reported snacking.

**Table 6:** Lifestyle and cardiometabolic characteristics of PREDIMED-Plus participants at baseline according to tertiles of the sQ-HPF score

	Tertiles of the sQ-HPF score			
	T1 (lowest)	T2	T3 (highest)	p^1^
**n**	1765	1223	1412	
**Weight (kg)**	83.32 (12.31)	87.07 (13.07)	90.68 (13.10)	**< 0.001**
**Waist circumference (cm)**	105.92 (9.66)	107.92 (9.61)	109.83 (9.45)	**< 0.001**
**BMI (kg/m**^**2**^**)**	32.60 (3.50)	32.63 (3.43)	32.53 (3.38)	0.699
**Glucose (mg/dL)**	114.12 (29.86)	114.98 (29.89)	112.94 (29.48)	0.098
**Total cholesterol (mg/dL)**	198.59 (38.18)	195.77 (37.63)	197.35 (38.78)	0.086
**Triglycerides (mg/dL)**	146.33 (72.04)	153.74 (76.26)	161.05 (90.55)	**< 0.001**
**Sleep time (hours/day)**	7.00 (1.24)	7.04 (1.23)	7.09 (1.16)	0.142
**TV time (hours/day)**	3.72 (1.82)	3.89 (1.92)	4.05 (2.02)	**< 0.001**
**Sedentariness (%)**	717 (40.6)	549 (44.9)	727 (51.5)	**< 0.001**
**Binge eating (%)**	114 (6.5)	80 (6.5)	119 (8.4)	0.066
**N° binges/week**^a^	2.37 (1.73)	2.48 (1.83)	2.6 (1.81)	0.613
**Snacking (%)**	469 (26.6)	326 (26.7)	430 (30.5)	**0.029**

Data shown as “mean (standard deviation, SD)” for continuous variables and as “number of subjects (%)” for categorical variables. One-way ANOVA test used for continuous variables and Chi-squared test used for categorical variables. Significant *p*-values (< 0.05) shown in bold

*BMI* Body Mass Index, *PA* Physical activity

^1^For continuous variables, *p*-values are adjusted for age, sex, recruitment center, energy intake, physical activity level, medication for blood pressure and diabetes, working status, educational level, and civil status

^a^Number of binges per week is calculated for those who answered “yes” to the question “binge eating” (overall *n* = 313, n by tertiles: T1 = 114, T2 = 80, T3 = 119). *N* = 4400

### Assessing longitudinal changes in HPF consumption with the sQ-HPF

Longitudinal analysis of HPF consumption estimated from the FFQ by each classification system revealed significant differences across the first 3 years of the PREDIMED-Plus study. In all cases, mean HPF consumption showed a trend towards a decrease that was lower in year 2 than baseline (Table 7). This was also the case when HPF consumption was estimated through the sQ-HPF (22.9 ± 0.22% of g/day at baseline and 17.3 ± 0.22% of g/day in year 2).

**Table 7:** Longitudinal changes in HPF consumption by PREDIMED-Plus participants

Study timepoint	Baseline	Year 1	Year 2	p for trend
**sQ-HPF score**	4.15 (0.04)	3.01 (0.04)	3.02 (0.04)	**< 0.0001**
**Quest. HPF (% of g/day)**	22.9 (0.22)	17.2 (0.22)	17.3 (0.22)	**< 0.0001**
**NOVA HPF (% of g/day)**	7.98 (0.12)	5.45 (1.12)	5.45 (0.12)	**< 0.0001**
**IARC HPF (% of g/day)**	45.6 (0.23)	40.3 (0.23)	39.9 (0.23)	**< 0.0001**
**IFIC HPF (% of g/day)**	20.1 (0.21)	16.8 (0.21)	16.4 (0.21)	**< 0.0001**
**UNC HPF (% of g/day)**	19.5 (0.22)	14.7 (0.22)	14.8 (0.22)	**< 0.0001**

Data are shown as “adjusted mean (standard error, SE)”. Linear mixed model used to assess changes in HPF consumption over time, adjusted for age, sex, recruitment center, energy intake, physical activity level, medication for blood pressure and diabetes, working status, educational level, and civil status. Significant linear trend *p*-values (< 0.05) shown in bold. HPF consumption was estimated as the percentage over total grams per day, except for fried foods (data not available). *N* = 3284

## Discussion

This article presents the sQ-HPF, a short, integrative, and easy-to-use questionnaire to estimate HPF consumption (Table 4). The development process involved a carefully designed statistical analysis and practical considerations for its use in a clinical/epidemiological context. This new tool includes 14 food and beverage items of which the frequency of consumption is recorded, based on a previously validated FFQ from the PREDIMED-Plus Trial [38–40]. Each item is scored as 1 if the frequency of consumption corresponds to the HPF dietary pattern, according to the calculated thresholds, and as 0 otherwise. Therefore, the higher the score, the higher the consumption of HPF. Moreover, this score can be used to estimate the percentage of HPF consumption over total intake, avoiding the need to administer a lengthy FFQ (Table 4) [63].

The present work demonstrates that statistical approaches such as EFA and Cronbach’s analysis are valuable tools for developing integrative tools related to dietary patterns and eating habits. Indeed, analysis of questionnaire items through EFA demonstrated that they identified one core construct, the HPF dietary pattern (Table 3) [64]. This was expected considering that items were selected based on a positive association with the variable tertile agreement through binomial logistic regression (Table 1). This resulted in 14 questions based on the frequency of consumption of food groups associated with a higher HPF consumption. One of the reasons for this was that, in this way, the questionnaire could identify people with an HPF dietary pattern with a focus on the frequency of consumption and not on the specific HPF items they consumed, so that the questionnaire could detect different HPF dietary patterns. Another reason for the selection of items is that the questionnaire can also provide information on how to intervene to improve health in other populations by identifying the food groups most frequently consumed by the respondent and allowing for customized targeting of the dietary intervention to reduce consumption of those food groups. Finally, this selection was performed to simplify the use and interpretation of the questionnaire. In this way, the questionnaire rapidly results in a score that increases with HPF consumption and can be used to categorize the respondent accordingly. Notably, the questionnaire was designed to encompass criteria from four leading food processing-based classification systems – NOVA, IARC, IFIC, and UNC –, so the categorization as HPF consumer occurs according to at least two of these classification systems (tertile agreement) [20]. This is a key asset of the sQ-HPF, which makes it more comprehensive than other methods that have been proposed and are limited to one classification system [63]. In this regard, the limitations of the NOVA system, the most used classification system, have been widely discussed [65] and it has been shown that the choice of the classification system can have a significant impact on research outcomes [20]. Therefore, the integrative approach used to create the sQ-HPF is undoubtedly a critical advantage contributing towards an objective measurement of HPF consumption and its association to disease. In addition, a reason for choosing food groups rather than specific food items was that this would allow assigning specific food items to groups in the questionnaire - even if they were not initially considered because they were not present in the FFQ - attending to similar characteristics than one of the 14 food groups.

Another significant advantage of the sQ-HPF is its quick and easy administration and interpretation, regardless of the method of administration (i.e., self-reported or interviewer-led). This is particularly useful at the epidemiological level, where a high volume of responses is often collected and the length and subsequent processing of questionnaires is an important aspect of study design [66]. Despite its apparent simplicity, the ability of the sQ-HPF to classify subjects according to their HPF consumption is comparable to other more involved classification systems (Supplementary Table 2). As a reflection of this, longitudinal changes in HPF consumption estimated by the four classification systems were also detected with the same significance by the sQ-HPF estimation (Table 7). Furthermore, significant differences among participants grouped according to sQ-HPF scores could be detected in weight, waist circumference, and hours spent watching TV, among others (Table 6). These results indicate the potential of the sQ-HPF as an initial tool to evaluate the impact of HPF consumption on lifestyle in large-scale intervention trials, such as PREDIMED-Plus. For these reasons, we firmly believe that the consistent use of the sQ-HPF in epidemiological studies could contribute to a robust understanding of the relationship between HPF dietary patterns and health.

The applicability of the sQ-HPF spans not only epidemiological research but also public health and clinical nutrition. The use of *“*a priori*”* screening tools to define dietary habits and patterns plays an important role in developing personalized nutrition advice [67]. Several epidemiological studies have shown the importance of using dietary patterns to assess the association between dietary exposure and risk of developing chronic diseases [14], giving rise to valuable screening tools aimed at large populations [68]. Most of the developed scores and indices emphasize the importance of healthy dietary habits – Mediterranean diet [41, 69], Nordic diet [70], “healthy” diet [71, 72] – based on a theoretical rationale. However, some studies have reflected the need to capture deleterious eating habits that present a counteractive distortion over the beneficial effects of these diets [67]. In this regard, some approximations focused on the assessment of positive health effects of the diet have included potentially harmful items in their composition, such as the Healthy Eating Index (HEI) [73, 74] and the Alternate HEI (AEHI) [75] to encapsulate the global adequacy to dietary guidelines. The sQ-HPF presented here could thus be a complementary tool that counteracts the analysis from questionnaires aimed at healthy dietary effects. In addition, its simplicity makes it easy to be implemented in the clinical practice, for example, at the level of primary care, for disease-preventing purposes. Furthermore, the sQ-HPF integrates the divergent UPF and HPF definitions through an agglomerative statistical analysis of commonly used criteria, which provides additional consistency to its application.

Regarding public health applications, the limitation of the so-called “discretionary foods” consumption has been gradually incorporated into health policies [76–78]. These foods include confectionery, soft drinks, biscuits, snacks, cakes and pastries, among others [76], most of which are considered HPF [30]. In keeping with this, we propose the use of the sQ-HPF as a screening tool to identify trends in consumption of these types of foods in large populations [79], to consistently identify foods that can be targeted in public health campaigns [19]. This will also contribute to harmonizing the methods of assessing government policies addressing the healthiness of food environments, a well-exposed need for the worldwide prevention of diet-related diseases [11, 19, 80].

One of the main limitations of the ad hoc development of the sQ-HPF is that its reproducibility in other populations is necessary. The PREDIMED-Plus cohort comprises older adults with metabolic syndrome and overweight or obesity and a limited HPF consumption, so the reproducibility of the sQ-HPF in populations with different characteristics will require further validation. Another limitation is that the FFQ used for the initial development of the questionnaire is not specifically designed to measure HPF consumption. However, it contains a considerable number of items categorized as HPF and UPF by current food processing-based classifications [20]. These include industrial biscuits, milkshakes, bakery products, cured meats, RTE preparations, snacks, and soft drinks. Therefore, the sQ-HPF was constructed so that specific HPF are very likely to fall into one of the groups defined in the questionnaire due to their similar description and characteristics to the items already specified in the questionnaire. The use of an FFQ has other limitations, such as recall bias that can impact the accuracy of the data collected [40], but its use to evaluate dietary intake in large epidemiological studies is widely recognized as suitable [39]. We aimed to develop a screener for HPF consumption, so it could be used as a tool that allowed rapid screening of a large population and the identification of consumers with a high HPF dietary profile. For an in-depth study of the specific items that make up the dietary pattern of each consumer, it will still be necessary to use more detailed FFQs or dietary recall interviews. Different research teams have developed FFQs aimed to evaluate dietary intake from HPFs [81, 82], so it would be of interest to compare these to the sQ-HPF through intra-class correlation coefficient analyses. In the case of a good correlation, we think that the sQ-HPF would present the advantage of being shorter and more direct regarding the estimation of HPF consumption, which will facilitate the work in epidemiological research and clinical practice.

## Conclusions

The sQ-HPF is an integrative, short, and easy-to-use questionnaire that can be used to screen HPF consumption in large populations for epidemiological or public health purposes. Due to the straightforward score calculation and interpretation, it could aid Personalized Nutrition practice and food policy-makers in the implementation of effective strategies to tackle diet-related disorders associated with HPF consumption.

## Supplementary Information

**Additional file 1.**

**Additional file 2.**

## Data Availability

There are restrictions on data availability for the PREDIMED-Plus trial due to the signed consent agreements around data sharing, which only allow access to external researchers for studies following the project purposes. Requestors wishing to access the PREDIMED-Plus trial data used in this study can make a request to the PREDIMED-Plus trial Steering Committee chair: jordi.salas@urv.cat. The request will then be passed to members of the PREDIMED-Plus Steering Committee for deliberation.

## Abbreviations

er-MedDiet: Energy-restricted Mediterranean diet
FFQ: Food frequency questionnaire
HPF: Highly processed food
IARC-EPIC: International Agency for Research on Cancer - European Prospective Investigation into Cancer and Nutrition
IFIC: International Food Information Council
MetS: Metabolic syndrome
NHS: Nurses’ Health Study
PA: Physical activity
PREDIMED: from the Spanish “PREvention with MEDiterranean DIet”
sQ-HPF: Screening questionnaire of HPF consumption
UNC: University of North Carolina
UPF: Ultra-processed food
