# Supplementary material for: Integrative development of a short screening questionnaire of highly processed food consumption (sQ-HPF)
Source: Int J Behav Nutr Phys Act. 2022 Jan 24;19:6. doi: 10.1186/s12966-021-01240-6 (PMC8785596; doi:10.1186/s12966-021-01240-6)
Supplement: Supplementary file 1 — Additional file 1. [file 12966_2021_1240_MOESM1_ESM.docx]

| **Supplementary Table 1. PREDIMED-Plus Food Frequency Questionnaire (FFQ) items included in each food group.** | |
| --- | --- |
|  |  |
| **FOOD GROUP** | **ITEMS** |
| **Whole dairy products** | whole milk, whole-milk yogurt, cottage cheese, curd, fresh cheese |
| **Semi-skimmed dairy products** | semi-skimmed milk, skimmed milk, skimmed-milk yogurt |
| **Fatty dairy products** | cream, cured or semi-cured cheese, processed soft cheese wedges |
| **Sugary dairy products** | condensed milk, industrially produced milkshakes, flavored Petit Suisse yogurt, custard, crème caramel flan, pudding |
| **Eggs** | eggs |
| **Red meats** | beef meat, pork meat, lamb meat, liver, offal, burger |
| **White meats** | skin-on chicken, skinless chicken, rabbit meat |
| **Cured meats** | serrano ham, sandwich (deli) ham, cured cold meats, pâté, bacon, marbling |
| **Fats** | margarine, butter, lard (animal fat) |
| **White fish** | white fish, oysters, mussels and clams, salted cod, calamari, octopus, baby cuttlefish, shrimp, prawn, lobster |
| **Blue fish** | blue fish, canned fish in water, canned fish in oil |
| **Vegetables** | chard, spinach, mushrooms, cabbage, cauliflower, broccoli, lettuce, chicory (endive), escarole, tomato, carrots, pumpkin, squash, flat green (Romano) beans, zucchini, eggplant, cucumber, pepper, canned white asparagus, gazpacho (cold Spanish tomato soup), other vegetables, onions, garlic, olives |
| **Fruits** | oranges, clementines, grapefruit, bananas, apples, pears, strawberries, cherries, plums, peach, apricot, nectarine, watermelon, melon, kiwi, grapes, raisins, natural orange juice, fruit juice |
| **Potatoes** | homemade French fries/chips, boiled/baked potatoes |
| **Nuts** | toasted almonds, salted pistachios, walnuts, salted/fried nuts |
| **Legumes** | lentils, dried beans, chickpeas, peas and beans |
| **Oils** | olive oil, extra virgin olive oil, olive pomace oil, corn (maize) oil, sunflower oil, soybean oil, mixed oil |
| **Fermented alcohols** | rosé wine, muscatel wine, young red wine, aged red wine, white wine, Spanish sparkling wine (cava), beer |
| **Distilled alcohols** | liquors, anisette, whisky, gin, vodka, cognac |
| **Non-sugary drinks** | decaf coffee, coffee, tea |
| **Sugary and artificially sweetened drinks** | soft drinks, artificially sweetened drinks, bottled juice, grape must |
| **Supplementary Table 1. *(Continued)*** | |
| **FOOD GROUP** | **ITEMS** |
| **Sweets** | ice-cream and sorbets, canned fruit in juice or syrup, biscuits, whole meal biscuits, chocolate biscuits, honey, homemade baking products, industrially produced confectionery, donuts, muffins, cupcakes, industrially produced cakes, churros, chocolates, soluble cocoa powder, nougat, marzipan, shortbread biscuits, jam |
| **Snacks** | packaged potato crisps/chips, packaged snacks |
| **Ready To Eat (RTE) products** | pizza, croquettes, instant soup |
| **Refined cereals** | white and sliced bread, breakfast cereals, spaghetti, macaroni, Spanish noodles, white rice |
| **Wholegrain cereals** | whole meal bread, muesli, whole meal cereals, whole meal pasta, whole meal rice |
| **Sauces** | mustard, mayonnaise, tomato sauce, Ketchup |
| **Additives** | sugar, table salt |
| **Fried foods** | eat out and homemade fried foods |

| **Supplementary Table 2. Agreement between tertiles of HPF consumption by the four classification systems and the sQ-HPF.** | |
| --- | --- |
|  |  |
| **sQ-HPF tertiles *vs.*:** | **Weighted Κ** |
| **NOVA tertiles** | 0.36 |
| **IARC tertiles** | 0.50 |
| **IFIC tertiles** | 0.65 |
| **UNC tertiles** | 0.82 |
| N = 4400. | |
